# Pivoting: from academic 3D printing to rapid COVID-19 solutions

**DOI:** 10.2217/3dp-2020-0022

**Published:** 2020-12-01

**Authors:** Allison R Powell, Charles A Keilin, Ross E Michaels, Owen J Tien, Kyle K VanKoevering, Glenn E Green, David A Zopf

**Affiliations:** 1 ^1^University of Michigan Medical School, Ann Arbor, MI 48109, USA; 2 ^2^MakeMedical, LLC, Ann Arbor, MI 48103, USA; 3 ^3^Department of Otolaryngology – Head & Neck Surgery, Ohio State University, Columbus, OH 43210, USA; 4 ^4^Department of Otolaryngology – Head & Neck Surgery, University of Michigan, Ann Arbor, MI 48109, USA; 5 ^5^Department of Biomedical Engineering, University of Michigan, Ann Arbor, MI 48109, USA

**Keywords:** additive manufacturing, computer-aided design, COVID-19, SARS-CoV-2

When a novel coronavirus (then known as 2019-nCoV) was first recognized in China in December 2019 [1], no one knew how devastating and widespread the COVID-19 pandemic would become. Across the world, this virus has overwhelmed modern healthcare systems in a way most could never have imagined. The challenges faced by physicians and healthcare systems have required rapid, innovative approaches to maximize safety for our patients and colleagues. In this brief Editorial, we describe how we repurposed our academic 3D printing laboratory in order to create operational patient care solutions that were safe and effective, in an attempt to address significant needs identified by healthcare providers at Michigan Medicine during the COVID-19 pandemic response. We hope that sharing our experience will be of use to other academic labs looking to make a similar transition in order to tackle the COVID-19 pandemic.

Before COVID-19, our academic lab used computer-aided design and 3D printing to research and develop medical simulators, patient-specific OR models, tissue engineering constructs and implanted devices like the 3D-printed tracheal splint [2]. Our multidisciplinary team, consisting of physicians and engineers working closely with business collaborators, made us uniquely prepared to rapidly mobilize our resources and shift our focus as the demands of the health system changed.

Central to our capacity to adapt to critical needs during the COVID-19 crisis are: the rapid manufacturing capacity of 3D printing; our robust network of physicians with real-time knowledge and exposure to clinical needs, and; our design/manufacturing expertise. The pandemic forced us to consider how we could repurpose our skills and leverage our experience and expertise to address the needs of our institution, region, state and nation.

Beyond our core team, we identified end users, including critical care and anesthesia providers and incorporated their feedback into our designs. The outpouring of willing supporters and collaborators was unprecedented and supported by the network of a large academic institution. In March, our institution accelerated research related to the pandemic by creating a specific review committee for such work [3], and we worked closely with our institutional review board and the US FDA to provide appropriate safety and improve timeliness to approval. Finally, we connected with business experts, medical manufacturing partners and distributors, so that we would be able to create and mobilize devices to the hospitals and patients in need.

To maximize impact, we conducted a brief needs assessment before beginning development and design. This helped us identify problems that were critical for patient care and also fit our skillset. We focused on three core problems: the potential shortage of mechanical ventilation equipment; the potential shortage of appropriate personal protective equipment, and; insufficient nasal swabs for COVID-19 testing.

We utilized an iterative design process similar to the quality improvement Plan–Do–Study–Act framework, which emphasizes multiple revisions before reaching a final product. Initially, simple sketches and computer-aided design renderings of the design helped team members and collaborators visualize the concept and provide feedback. 3D printing was used for rapid prototyping, which reduces turnaround between designs from days to hours. This technology also utilizes inexpensive alternative materials during the design-phase, allowing it to be used efficiently for rapid iteration.

Our team addressed the three core problems simultaneously. Within 2 weeks from idea conception, we created and built a mechanical ventilator splitting circuit that attaches to a single ventilator to provide tailored, individualized pressures to two or more patients simultaneously. 3 weeks later, after appropriate preclinical testing, FDA Emergency Use Authorization was obtained with the support of several regulatory specialists. We saw similar success with protective face shields, which went from concept to mass manufacturing through our partnership with a local 3D printing company within 1 week. We recruited medical students to assist in preparing headbands, which allowed production of over 10,000 protective face shields for healthcare workers that have been distributed in our and neighboring hospital systems. When a shortage of nasal swabs was projected at our institution, a collaboration was formed between our lab team and Formlabs (MA, USA) to expedite in-house production of medical grade nasal swabs, resulting in the capacity to manufacture over 2000 swabs per day, if necessary.

It is important to recognize that while 3D printing allows for faster iteration and development, purpose and safety must remain paramount. Instead of racing to design devices that may not solve a problem, we began by completing a realistic needs assessment. Only once we identified immediate problems that we were equipped to solve, did we proceed. We also took every precaution to keep essential lab members safe. Much of our work can be done virtually, and we restricted the number of members working in the laboratory spaces for safety reasons, in accordance with our institution’s recommended guidelines. While collaboration is crucial to developing a safe and effective device, it is not always easy. The pandemic has made communication more difficult by limiting in-person contact, and it was important to maintain effective communication through daily virtual meetings. This ensured that the core team continued to work together with a consistent goal in mind. Finally, we remained mindful of patient and provider safety. Our lab is tailored for innovative design and translational device development, but it is not an International Organization for Standardization-compliant manufacturing facility. To ensure the quality of the end product, we partnered with established medical manufacturers for each device we designed. In addition, while the FDA has accelerated Emergency Use Authorization for critical device needs in this pandemic environment, adequate preclinical testing and ongoing clinical evaluations of devices in use is underway.

COVID-19 has challenged our ability to care for patients in many ways, but academic institutions are uniquely positioned to bring together experts with multidisciplinary collaboration to overcome challenges, even in these times. Our team has long employed this interdisciplinary approach, and as an academic research lab, we were able to redirect our resources to address the rapidly changing needs of patients and caregivers during the pandemic. Identifying problems that we had the skills to solve, and collaborating with a range of stakeholders, within and beyond academia, was essential to develop functional and scalable designs and successfully reach those in need safely and efficiently. We believe this approach represents an untapped opportunity for innovation in academic medicine, and we challenge other laboratories to identify and repurpose their unique skills to meet the evolving needs of this pandemic and beyond.
